# Tolerance Induction in Relation to the Eye

**DOI:** 10.3389/fimmu.2018.02304

**Published:** 2018-10-09

**Authors:** Igal Gery, Rachel R. Caspi

**Affiliations:** Laboratory of Immunology, National Eye Institute, Bethesda, MD, United States

**Keywords:** experimental autoimmune uveitis (EAU), ocular inflammation (uveitis), tolerance process, T-helper (Th) cells, T-regulatory cells (Treg), microbiota

## Abstract

Inflammatory intraocular eye diseases, grouped under the term uveitis are blinding conditions, believed to be mediated by pathogenic autoimmune processes that overcome the protective mechanisms of the immune privilege status of the eye. An animal model for these diseases, named experimental autoimmune uveitis (EAU), is induced by initiation of immunity against ocular-specific antigens, or it develops spontaneously in mice with T-cells that transgenically express TCR specific to the target eye antigen(s). T-Cells specific to ocular antigens are generated in the thymus and their majority are eliminated by exposure to their target antigen expressed in this organ. T-cells that escape this negative selection acquire pathogenicity by their activation with the target antigen. In spontaneous EAU, the microbiota play crucial roles in the acquisition of pathogenicity by providing both antigenic stimulation, by molecules that mimic the target ocular antigen, and an additional stimulation that allows invasion of tissues that harbor the target antigen. The pathogenic process is physiologically inhibited by the peripheral tolerance, composed of antigen-specific T-regulatory (Treg) lymphocytes. Deleting the Tregs enhances the ocular inflammation, whereas adoptively transferring them suppresses the pathogenic response. Potential usage of Treg cells for suppression of autoimmune diseases in humans is under intensive investigation.

## Introduction

The clarity of ocular tissues and spaces is crucial for sight. Inflammatory processes thus have detrimental effects on vision, by causing media opacity. Furthermore, the damaging effects of inflammation in the eye are also harmful by affecting photoreceptor cells that are critical for the process of vision, and do not regenerate. To protect against immune-mediated inflammatory processes, the eye has evolved multiple layers of defense that endow it with a status of immune privileged organ (1). These layers of defense, studied, and defined by many investigators over the course of recent four decades include: (i) an efficient blood-retina barrier; (ii) an immunoinhibitory environment composed of several soluble and cell-bound immunosuppressive molecules; (iii) an active process known as “anterior chamber associated immune deviation (ACAID)” which can be demonstrated experimentally, in which foreign antigens reaching the anterior chamber of the eye initiate a complex immunosuppressive process (2, 3).

Early on, it was thought that due to their sequestration in the eye, ocular antigens, in particular those of the retina, are treated as foreign and provoke immune responses when exposed to the immune system. This notion was supported by the features of Sympathetic Ophthalmia, a disease in which trauma to one eye is followed by inflammation in the other, undamaged, eye. Autoimmunity has been proposed to play a major role in the pathogenesis of sympathetic ophthalmia, as well as in the pathogenesis of other human eye diseases in which intraocular inflammation without an accompanying infection, is the major symptom. These include birdshot chorioretinopathy, Behcet's disease, Sarcoidosis, and Vogt-Koyanagi-Harada (VKH) disease; other inflammatory eye diseases listed under the “uveitis” umbrella are not covered here.

Pathogenic autoimmunity is also assumed to be the mechanism responsible for phacoanaphylactic endophthalmitis, a rare but severe eye disease that follows damage to the lens and the release of lens antigens, proteins that are normally sequestered from the immune system (4).

As will be discussed below, the “physiological” approach to treating autoimmune-mediated diseases would be by induction of specific tolerance to the autoantigens that are the targets for the pathogenic response. We summarize here basic data concerning these issues, specifically dealing with tolerance to antigenic components of intraocular tissues of the eye. Immunity and tolerance to the cornea and external eye tissues have been investigated thoroughly and were reviewed elsewhere (5–7).

## Uveitis—current status

Intraocular inflammatory conditions in humans are grouped under the term noninfectious uveitis, that includes the five eye diseases mentioned above (8, 9). Autoimmune processes are believed to play a major role in the pathogenesis of these diseases, a notion supported by findings of cellular and/or humoral immune responses against ocular auto-antigens in these patients (8–11). In addition, involvement of autoimmune processes is strongly supported by the similarity between the pathological changes specific to these human diseases and the ocular changes seen in animals with experimental autoimmune uveoretinitis (EAU) (8, 9, 12). This animal disease is induced by immunization with retina-specific antigens and is mediated by pathogenic T- lymphocytes (9, 13, 14). The notion that the human conditions are mediated by immunopathogenic T-cells is also supported by the finding that, like animal models (15, 16), patients with these conditions tend to respond positively to treatment with immunosuppressive agents that mainly target T cells, such as cyclosporine, rapamycin, and FK-506 (16–18) and, more recently, by daclizumab (antibody to the interleukin 2 receptor) and other biologics (19–21).

Treatment with immunosuppressive agents, however, inevitably has adverse effects on host defense. Therefore, the ideal treatment would be induction of antigen-specific tolerance. Selective tolerance to uveitogenic antigens has been achieved in animals, in which treatment with a uveitogenic protein (S-antigen/retinal arrestin) yielded inhibition of EAU induced by immunization with this same molecule (22, 23). In these studies the antigen was administered by oral gavage during the development of the experimental disease and Dick et al. (24) reported high efficiency of EAU suppression by intranasal administration of the retinal antigen. Since patients with uveitis often demonstrate immunity toward retinal S-antigen (10, 25), studies were carried out to examine the efficacy of treating such patients for induction of oral tolerance by feeding with this antigen (26), or with an HLA peptide that crossreacts with S-antigen (27). The initial results appeared encouraging, but additional studies are required to examine the therapeutic usefulness of the procedure in more depth. The main advantage of oral tolerance is the absence of known adverse effects, but its drawback, as with other antigen-specific tolerance approaches, is that it requires knowledge of the inciting antigens. However, the specific antigens that serve as molecular targets for uveitis are not certain. That said, the phenomenon of tissue specific bystander suppression, where regulatory cells induced to one antigen may suppress responses to other antigens in the same tissue environment (28) could, in theory, be exploited to get around this limitation.

## Central and peripheral tolerance in relation to the eye

Over the past few decades, research with animals, primarily mice, has provided a large amount of information concerning the complex process of tolerance. The state of tolerance to self antigens is achieved by two separate processes: central and peripheral tolerance, which operate in tandem. When either of these fails, autoimmunity can result. Central tolerance occurs in the thymus. As part of their development, new T-lymphocytes with specificity toward a wide range of antigens are generated in the thymic cortex, and follow a stereotypic process of maturation through several well defined stages (29, 30). As part of this process, maturing T-cells with high affinity toward autoantigens undergo apoptotic death in the thymic medulla upon exposure to their cognate tissue antigens, whereas T cells with intermediate affinity may be converted into natural T regulatory cells (nTregs). The tissue antigens that trigger this process of thymic education were shown to be expressed ectopically by thymic medullary epithelial cells (29, 30).

Several groups have examined the process of central and peripheral tolerance toward retinal antigens, using transgenic mice expressing a neo-self antigen in the retina (hen egg lysozyme = HEL, or β-galactosidase = β-Gal) and/or a transgenic TCR to the neo- or a native self-antigen (31–36). With one exception, which may have been due to technical limitations (32), the conclusion reached by these studies is that there was detectable elimination of retina-specific T cells in the thymus. This was subsequently shown to be due to expression of retinal antigens (or retinal neo-antigens) in the thymus under control of the AutoImmune REgulator (AIRE) transcription factor that drives expression of tissue-specific antigens, including retinal antigens, in the thymus, and mediates negative selection of autoreactive lymphocytes (37, 38). Expression in the thymus of a natural uveitogenic retinal antigen, interphotoreceptor retinoid-binding protein (IRBP), was demonstrated by using RT-PCR (39) and immunohistochemistry (36) and was positively correlated in different mouse strains with their resistance to EAU (39) and with elimination of reactivity to specific uveitogenic epitopes from the immune repertoire (40). Importantly, testing thymic expression of ocular-specific genes (S-antigen, IRBP, RPE65, and recoverin) in thymi of several human individuals revealed a remarkable variability in the level of expression of these molecules (41). These data thus provide a possible mechanistic explanation for the differences among individuals in their susceptibility to autoimmune uveitis and to tissue specific autoimmune diseases in general, suggesting that the susceptibility is regulated at least in part by the level of thymic expression of the pathogenic autoantigens.

It is also of note that AIRE-controlled expression of self antigens plays an important role in the generation of thymus-derived natural Treg cells (nTregs) (42, 43). This likely includes also IRBP-specific nTreg cells that might account for raising the threshold of susceptibility to EAU, even though after a uveitogenic challenge, disease may be regulated also by induced Tregs (43), elicited in the periphery as a result of the immunization and disease process.

The process of thymic central tolerance alone is insufficient to eliminate all the T-lymphocytes reactive to tissue-specific antigens and consequently, a proportion of self-reactive cells manage to exit to the periphery. Such escapee cells are normally kept in check by a process known as peripheral tolerance, namely, exposure to the cognate antigen in the tissue in the absence of costimulatory danger signals, precipitates immunological paralysis (anergy), or conversion to Tregs (dubbed peripheral or induced Tregs). In a curious way, this parallels the process of central tolerance, in that it requires contact with the cognate antigen, whereupon the autoreactive cells are disarmed.

**Figure 1:**
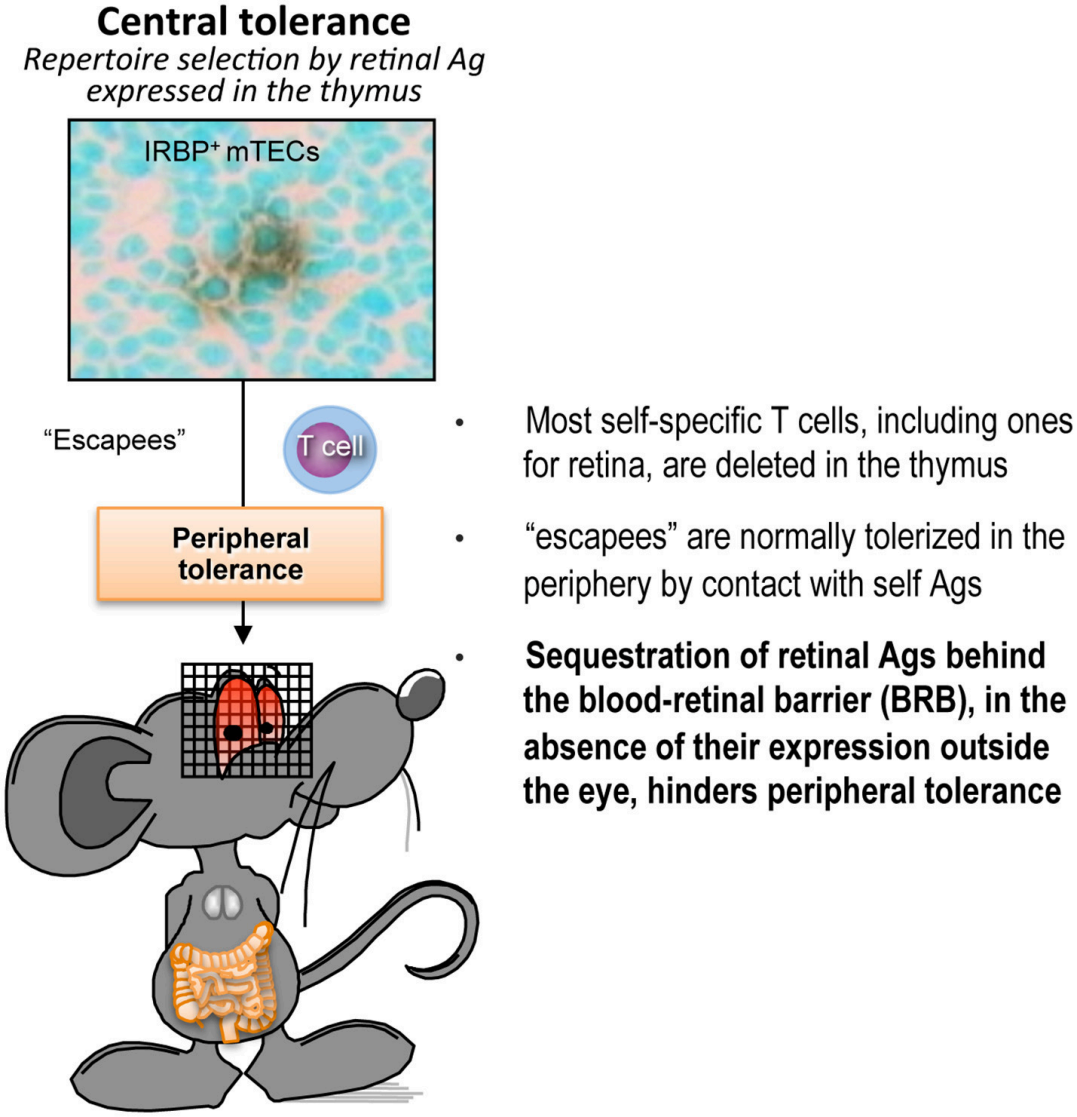
Peripheral tolerance is inefficient in the case of retina-specific T cells. Schematic representation of the process of self-tolerance to retinal antigens. Adapted from Horai and Caspi (48). No copyright permissions are required for the reuse of this image.

In the case of the eye, however, peripheral tolerance may not operate efficiently due to limited accessibility of the tissue antigens which are unique to the eye, and are largely sequestered behind a blood retinal barrier. In support of this notion, retinal neo-antigen presentation is not detectable in eye-draining lymph nodes, and circulating TCR Tg lymphocytes specific to retina display a largely naïve phenotype (31, 33, 44, 45). Therefore, we believe that T cells that have not been deleted in the thymus persist in the periphery in a non-tolerant state. This notion is further supported by data showing that gene expression of retinal antigens outside the eye, e.g., by transgenesis, retroviral transduction, or hydrodynamic injection, confers profound resistance to EAU (33, 43, 46). Furthermore, healthy humans have a relatively high frequency of circulating T cells specific to retinal antigens (47). These concepts are illustrated in Figure 1. In the aggregate, the data paint a picture whereby tolerance to retinal antigens is dependent mainly on thymic selection, whereas peripheral tolerance mechanisms are a “weak link” that may present an opportunity of therapeutic manipulation.

## Natural and experimental triggers of uveitis

Despite their presence in the circulation, naïve autoreactive T cells clearly do not cause uveitis in most individuals; perhaps, because they are unable to invade the target organs without additional activation processes. It has been well established in models of autoimmune disease that in order to elicit disease by infusion of autoimmune lymphocytes, these cells must first be activated *in vitro*. The notion that circulating naïve T cells are incapable of invading normal tissues, is supported by findings in mice. Thus, naïve CD4 T cells specific to a neo-self eye antigen do not induce ocular inflammation, unless they are pre-activated (*in vitro)* (49) and the same was true of T cells that express transgenic TCR specific for the natural retinal antigen IRBP (44). This leads to the question, where do retina-specific uveitogenic lymphocytes become activated *in vivo* to acquire the ability to penetrate the blood-retinal barrier and infiltrate the eye. Importantly, exposure *in vivo* to innate stimuli, such as TLR ligands, or complete Freund's adjuvant (which contains heat killed mycobacteria), without *in vitro* preactivation of the autoreactive cells, could be sufficient to support induction of uveitis in both uveitis models mentioned above (39, 40). While additional effects on the host, such as increased leakiness of the vasculature (50), could not be excluded as contributing factors, these observations strongly suggest that microbial stimuli might be involved in triggering uveitis.

**Figure 2:**
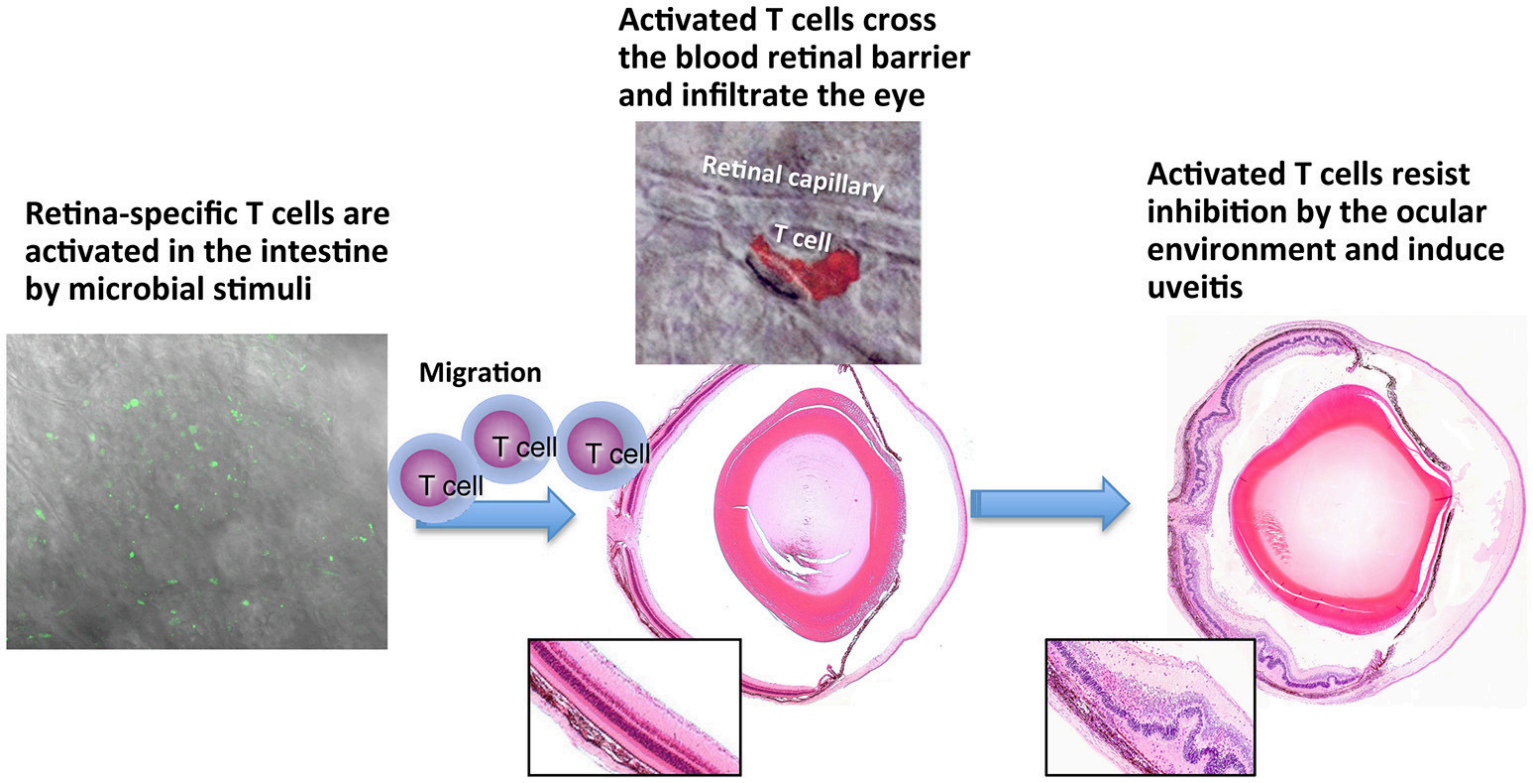
Persisting retina-specific T cells can be activated by peripheral stimuli. Schematic representation of EAU induction after activation of retina-specific T cells in the gut. Green cells in left panel are nurllgfP-positive T cells that are signaling through their T cell receptor [Horai et al. (45)]. Red cell in the middle top panel is a retina-specific T cell extravasating from a retinal vessel. The cell is stained by the PKH26 tracking dye and this photo was previously published in Prendergast et al. (50). No copyright permissions are required for the reuse of this image.

Extrapolating from the knowledge that innate microbial signals (e.g., complete Freund's adjuvant) drive tissue specific T cells to a pathogenic effector phenotype, Caspi and coworkers further explored the role of commensal microbiota as a trigger of uveitis. Toward that end, they developed the R161H mouse strain, which expresses an IRBP specific TCR and develop autoimmune uveitis spontaneously (44). Rearing the mice under cover of broad spectrum antibiotics, or under germ-free conditions, strongly attenuated development of disease, supporting the notion that commensal flora can serve as a trigger of autoimmune uveitis (45). Importantly, in microbe-containing mice, the retina-specific R161H cells were seen to signal through their IRBP-specific receptors in the intestine, suggesting that they were being activated *in situ* by commensal flora in an antigen specific fashion to trigger disease. The proposed scenario of the gut-eye axis in uveitis is depicted in Figure 2. Interestingly, Gery and colleagues found (51, 52), that the process of pathogenicity acquisition by uveitogenic T-cells requires an additional phase of 2–3 days in the spleen, and possibly other organs, during which *in vitro* activated autoimmune lymphocytes are “licensed” to invade tissues where the target antigen is located. Similar findings were reported for the brain, another immune-privileged tissue (53).

## Immune privilege and uveitis

As briefly discussed above, the eye is protected from the immune system by the complex phenomenon of immune privilege, in which sequestration is only the first layer of defense. The internal environment of the eye contains a variety of potent soluble and cell-bound inhibitory molecules, including TGF-β, α-MSH, CGRP, VIP, and retinoic acid, as well as FasL, PD-L1, TSP-1, to name a few [reviewed in ref (3)]. Multiple studies over the last 3 decades presented evidence that ocular fluids and ocular resident cells inhibit activation and function of various immune cells in culture, and can even convert T helper (Th) cells to Foxp3^+^ Tregs (54). How, then, can we explain induction of uveitis in the face of immune privilege? To examine whether the living eye has the ability to control activation of uveitogenic T cells, Caspi and colleagues injected IRBP-specific T cells obtained from R161H donor mice directly into eyes of recipient mice, and followed their fate. The data showed that naïve R161H T cells recognized their cognate antigen but did not acquire effector function in the eye. Instead, they converted to Foxp3^+^ Treg cells, which were functional and were able to inhibit activation of fresh R161H T cells in a standard T cell suppression assay (54). In contrast, antigen-experienced R161H T cells, isolated from IRBP immunized R161H mice by selection for activation/memory markers, that had been exposed to IRBP before being placed in the eye, could not be inhibited by the suppressive intraocular environment and caused extensive photoreceptor damage (54). This can explain why T cells that have been activated outside the eye—whether as a result of trauma or activation in the gut—and have acquired the ability to penetrate the blood-retinal barrier, can induce uveitis despite ocular immune privilege.

## Natural and therapeutic regulation of uveitis

Peripheral tolerance against pathogenic autoimmunity is executed by several types of regulatory cells, including, but are not limited to, natural, and induced Tregs. Treg cells are capable of killing and/or suppressing T-lymphocytes specific to autoantigens, that escaped the negative selection process in the thymus and migrated into the blood and lymphoid organs [reviewed in ref (55)]. In early studies, the CD4 Treg cells were identified mainly by their strong surface expression of CD25 (56), but their identification has been greatly improved by the finding that their majority express the transcription factor FoxP3. Depletion of Foxp3+ Tregs caused the mice to develop more severe uveitis upon IRBP challenge, indicating that preexisting (likely thymic-derived) Tregs raise the threshold of susceptibility to uveitis (57). In mice that already developed uveitis, depletion of Foxp3+ Tregs at the peak of disease prevented resolution, and depletion after resolution induced a relapse, indicating that Foxp3+ Tregs naturally bring about and maintain resolution of EAU (43). The suppressive activity of Treg cells in the EAU system was also examined by Gregerson group, who found that Tregs may be generated in the eye and protect the retina from EAU induced by active immunization or adoptively transferred pathogenic Th cells. Interestingly, the generation of local Treg cells is inhibited by selective elimination of dendritic cells (DC) and microglia cannot replace DC in this function (58).

Treg cells that do not express Foxp3 (Tr1) have also been reported and characterized (59). Tr1 cells may be important in uveitis, but less is known about them, as they are more difficult to identify, and therefore to study. Tr1 cells are induced by IL-27 (60), as well as by c-Maf, Il-21 and ICOS (61). Caspi group demonstrated that IL-27 is potently induced in DC by IFN-γ-producing natural killer (NK) cells, in a self-amplifying feedback loop that takes place in the draining lymph node of mice immunized for uveitis. This interaction dampens the immune response that leads to uveitis, thus identifying NK cells as a novel regulatory cell that controls the magnitude of the autoimmune response (62). Other cells identified to be immunosuppressive in the context of EAU are IL-35 producing B-cells that were recently found by Egwuagu and colleagues to suppress EAU development, in part through induction of IL-35-producing Tregs (63, 64). Interestingly, this group showed that the IL-12p35 subunit has immunoregulatory functions that were hitherto attributed to IL-35 (65). IL-35 is also expressed by Treg cells and, interestingly, Wei et al. reported that that different subpopulations of Tregs produce IL-35 or IL-10 (66).

Based on the data described above, the group of Caspi set out to examine whether therapeutic induction of tolerance could regulate the pathogenic process of EAU. Treatment of mice with an IRBP expression plasmid, in the form of naked DNA administered by hydrodynamic injection, markedly suppressed the induction of EAU in the treated mice and analysis of the inhibitory process revealed that the inhibition of EAU was mostly due to CD4+CD25+FoxP3+ regulatory cells (43). Notably, Treg cells from hydrodynamically injected mice could be expanded into functionally suppressive Treg cell lines that, when adoptively transferred to mice immunized with IRBP, inhibited EAU development in the recipient mice (43).

In view of the specific and potent inhibitory capacity of Treg cells, the notion of using these cells in suppression of autoimmune disease in humans seems very attractive (67). One approach is to isolate antigen-specific Treg cells, increase their numbers *ex vivo* and inject them back to the patient (68). Another approach, proposed by the Salomon group (69), offers uveitis as the model disease, and uses preactivated polyclonal Treg cells that would exert bystander suppression in the target tissue. The system was tested in mice with EAU and suppression of disease was achieved, but only when the cells were injected into the vitreous. So far, no data have been reported to show successful treatment of uveitis patients with Treg cells. However, the notion that Treg cells are involved in suppression of the pathogenic process of uveitis is supported by the finding of a correlation between increase in the proportions of Treg cells in blood of uveitic patients and remission of the disease (70). Furthermore, the frequency of Treg cells was found to decline in parallel with increase in the severity of the uveitic changes (71).

In addition to T and B cells, myeloid cells can also regulate EAU. Myeloid-derived suppressor cells (MDSC) are a rather heterogeneous myeloid cell population that may include monocyte/macrophage- and granulocyte-like populations. Evidence reported by several groups indicates that MDSC could act at the systemic as well as local levels to curb disease (72, 73). Importantly, MDSC have also been identified in association with human uveitis (73). Additionally, dendritic cells (DC) having a tolerogenic phenotype may also play a regulatory role. Forrester and collaborators reported that treatment of mice with LPS-induced tolerogenic DCs, which produced IL-2 and suppressed uveitis by multiple mechanisms (74).

Finally, ocular inflammation may also be regulated by non-lymphoid cells, known as mesenchymal stem/stromal cells (MSCs). The mechanisms may involve induction of MDSC-like cells (75) as well as induction of Treg cells (76).

## Author contributions

All authors listed have made a substantial, direct and intellectual contribution to the work, and approved it for publication.

### Conflict of interest statement

The authors declare that the research was conducted in the absence of any commercial or financial relationships that could be construed as a potential conflict of interest.
